# Supercritical water anomalies in the vicinity of the Widom line

**DOI:** 10.1038/s41598-019-51843-0

**Published:** 2019-10-31

**Authors:** Konstantinos Karalis, Christian Ludwig, Bojan Niceno

**Affiliations:** 1 Laboratory for Scientific Computing and Modelling (LSM), NES Division, Paul Scherrer Institute, 5232, Villigen PSI, Switzerland; 2 Laboratory for Bioenergy and Catalysis (LBK), ENE Division, Paul Scherrer Institute, 5232, Villigen PSI, Switzerland; 3 École Polytechnique Fédérale de Lausanne (EPFL), ENAC IIE GR-LUD, 1015 Lausanne, Switzerland; 4 Eidgenössische Technische Hochschule Zürich (ETHZ), MAVT-LKE, 8092 Zurich, Switzerland

**Keywords:** Atomistic models, Renewable energy

## Abstract

Supercritical water is used in a variety of chemical and industrial applications. As a consequence, a detailed knowledge of the structure-properties correlations is of uttermost importance. Although supercritical water was considered as a homogeneous fluid, recent studies revealed an anomalous behaviour due to nanoscale density fluctuations (inhomogeneity). The inhomogeneity is clearly demarked through the Widom line (maxima in response factions) and drastically affect the properties. In the current study the physical properties of supercritical water have been determined by classical molecular dynamics simulations using a variety of polarized and polarizable interatomic potentials. Their validity which was not available at supercritical conditions has been assessed based on the ability to reproduce experimental data. Overall, the polarized TIP4P/2005 model accurately predicted the properties of water in both liquid-like and gas-like regions. All interatomic potentials captured the anomalous behaviour providing a direct evidence of molecular-scale inhomogeneity.

## Introduction

Supercritical water (SCW) is of extreme importance both for fundamental research and industrial applications. SCW is a cheap inorganic and green (non-toxic) solvent as alternative to chemical (organic and toxic) industrial solvents, having thus a wide applicability in chemistry processes due to the singular physical and chemical properties^1–3^. More specifically, it is a very promising medium for various emerging chemical, biological and geological processes including chemical synthesis^4^, biomass processing^5–8^, hazardous treatment^9^ and carbon capture and storage^10^. Consequently, the complete understanding of the SCW behaviour for a wide range of thermodynamic conditions is essential.

A fluid can be characterized supercritical when the temperature and pressure are higher than the critical point (in the case of water T_c_ > 647.096 K and P_c_ > 220.640 bar). Beyond the critical point, no physically observable difference between a liquid and a gas exists and hence a single fluid-phase region is considered^11–13^. However, in this region, the correlation length and the thermodynamic response functions which are derivatives of the state functions with respect to temperature and pressure (e.g., isobaric heat capacity, isothermal compressibility and thermal expansion coefficient) have maxima defining lines emanating from the critical point, termed the “Widom lines”^13–15^. The critical anomalies on the Widom lines demarcates two regions, the liquid-like and the gas-like^15–19^. In the vicinity the critical point, Widom lines merge into a single line and this anomalous behaviour (maxima of the response functions) progressively vanish^20,21^ by creating a deltoid coexistence region^16^. Usually the locus of specific heat maxima is referred as the Widom line^22^.

In fluids, there is one more line as a dynamical crossover which is termed the Frenkel line^15^. The Frenkel line demarcates two regions in which the fluid behaves as non-rigid (dense gas-like behavior) and rigid-liquid (solid-like behavior)^15,23^. In the gas-like regime atoms have only diffusive motion while in the liquid-like regime atoms combine both solid-like quasiharmonic vibrational motion and gas-like diffusive motion^24^. The most convenient way to quantitatively determine the location of Frenkel line in the phase diagram is by calculating the velocity autocorrelation function (VAF) of the fluid (the disappearance of oscillation and minima of the VAF)^15,24,25^. In case of pure water at pressures smaller than 380 bar the Widom line can be used as the crossover line of dynamical properties while at higher pressures the Frenkel line should be used^15^.

Due to high SCW compressibility (directly related to microscopic density fluctuations^17^), by slightly adjusting the thermodynamic conditions, the structural, dynamical and transport properties drastically altered allowing thus the eclectic dissolution of polar or non-polar solutes^3,26–30^. This phenomenon is more intense in the vicinity of the critical point (1 < T/T_c_ < 1.1 and 1 < P/P_c_ < 1.2). The substantial density changes associated with the hydrogen bonding drastically affect the diffusivity, dielectric constant, viscosity and thermal conductivity thus influencing the mechanisms and kinetics of chemical reactions^28,30^. Even the existence of hydrogen bonds in SCW was in controversy, now it is generally accepted that hydrogen bonds are formed in supercritical conditions (in the non-high density states the hydrogen bonds are below the percolation threshold of 1.58), although hydrogen bonded networks do not exist^30–35^.

It has so far been proven that the physical properties can be resolved via classical molecular dynamics (MD) simulations using interatomic potentials (force-fields). The force-fields (FFs) which characterize the strength and the nature of interactions between atoms, are categorized in terms of bond rigidity/flexibility and polarizability^22^. EvC FFs, they have been mainly confined to study water at ambient conditions and consequently their validity at supercritical conditions needed to be verified. In this work, we extensively analysed (wide range of thermodynamic conditions) the SCW properties using MD simulations. The most widely used polarized (SPC/E and TIP4P/2005) and polarizable (BK3 and SWM4-NDP) FFs have been assessed based on their ability to reproduce experimental values at supercritical conditions. The Widom line was determined identifying the transition from liquid-like (LL) to gas-like (GL).

## Results and Discussion

The structural characteristics and physical properties of SCW for a wide range of temperatures (600–700 K) and pressures (230–290 bar) were determined by MD simulations and compared with water equation of state (EoS) provided by NIST^36^ and data obtained from IAPWS^37^. Based on the maximum of the heat capacity, using multiple FFs, the Widom line (distinguishing the liquid-like and gas-like SCW behaviour) was determined. Due to the fact that all water models fail to reproduce the experimental critical temperature and pressure, an offset for both parameters (see the Molecular Dynamics Method subsection) has been applied^38–40^.

### Bulk-density (ρ)

**Figure 1:**
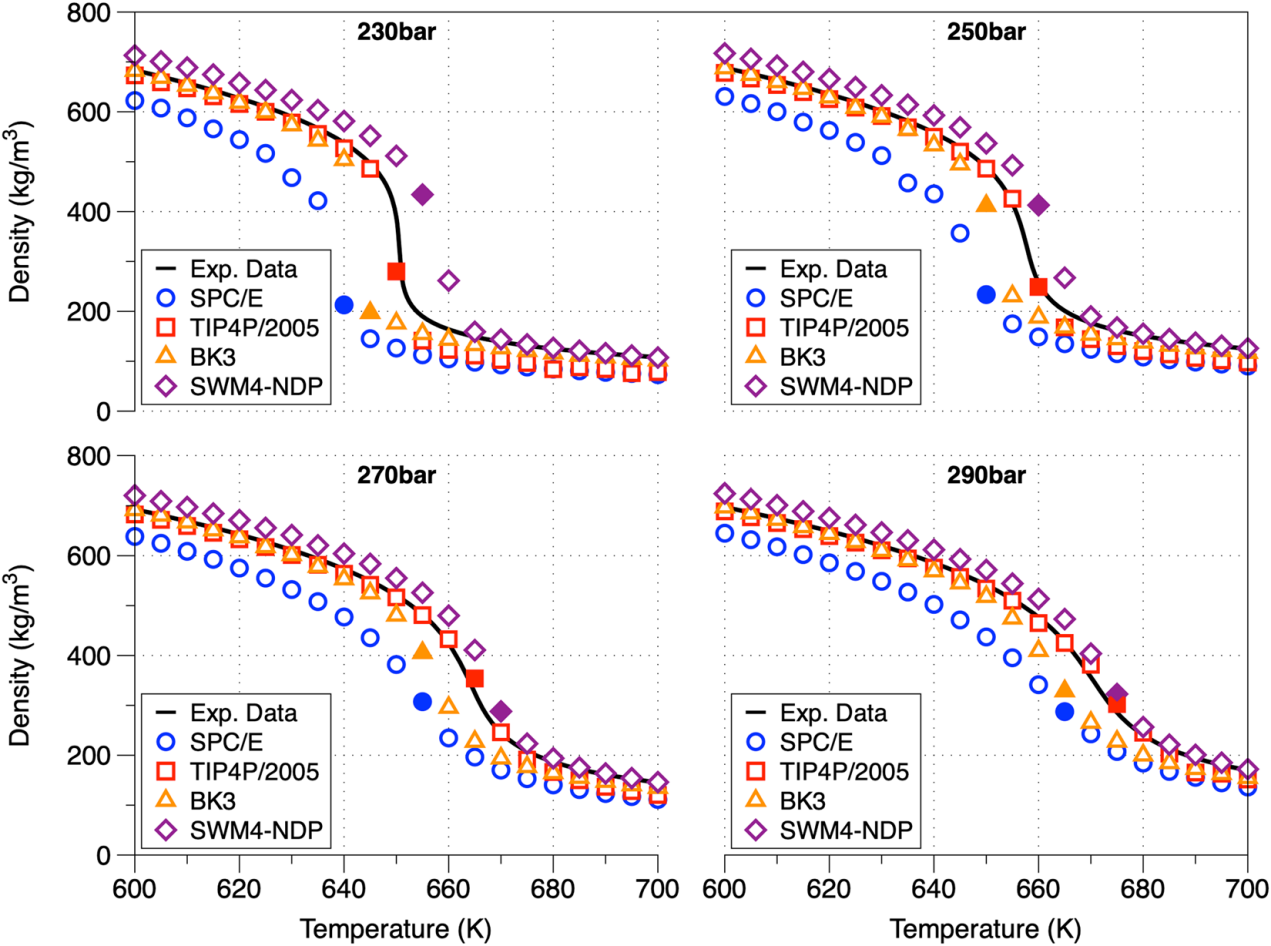
Supercritical isobars using different interatomic potentials (SPC/E, TIP4P/2005, BKE and SWM4-NDP) in comparison with experimental results. The filled symbols describe the temperature at which the maximum heat capacity line (point in the Widom line) is crossed (Supplementary Information). The Widom point signs the change of water behavior from liquid-like to gas-like. The black line refers to experimental data obtained using water EoS^36^.

**Figure 2:**
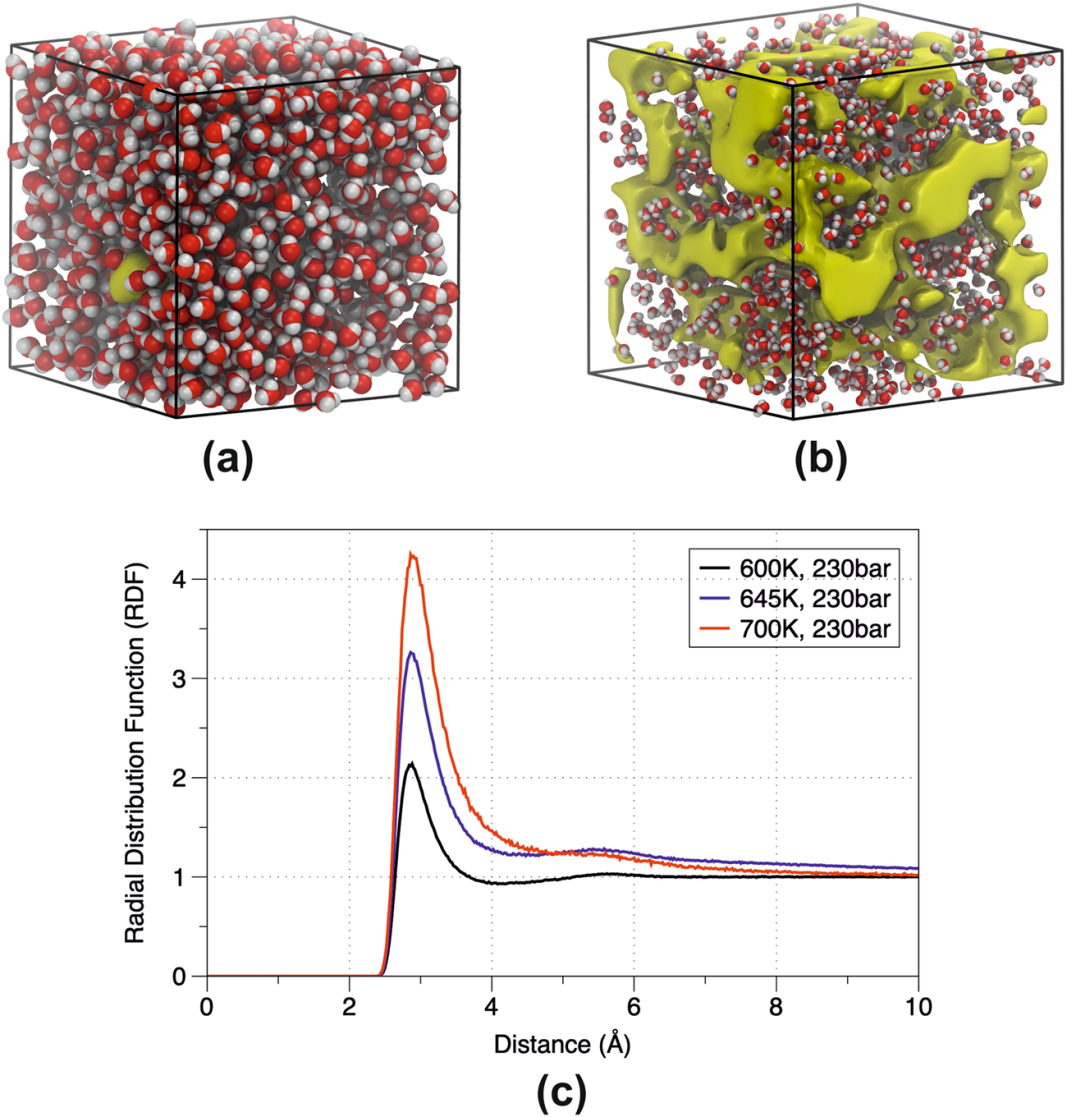
Water molecules and void isosurfaces (represent those points in space which all water molecules exhibit a distance more than 3 Å^62^) portraying the inhomogeneity in (**a**) the liquid-like phase and (**b**) the gas-like phase. The red color presents the oxygen atoms, the gray the hydrogen atoms and the yellow isosurfaces the voids inside the supercell. For the visualization the VMD code was used^63^. (**c**) Radial Distribution function (RDF) of Oxygen-Oxygen interactions in the liquid-like and gas-like phases in respect to different pressures using the TIP4P/2005 model.

The distribution of mass density along four isobars at different temperatures is shown in Fig. 1. The SPC/E model consistently underestimates the experimental data by approximately 10–15% in the low temperature regime (600–630 K) and 24–47% in the second half of the temperature range. This is attributed to the fact that SPC/E fail to resolve the critical density (273 kg/m^3^ instead of 322 kg/m^3^)^39^. The TIP4P/2005 model accurately predicts the densities and the inflection points across all isobars (provides a good description of the water phase diagram^41^) having almost a near-perfect agreement with the largest discrepancies at high temperatures. The BK3 model perfectly predicts the density in the low temperature regime (deviation smaller than 1%) while at higher temperatures underestimate the densities with a deviation of 5.5–10%. The higher deviation in the density calculation at higher temperatures is attributed to the increase of inhomogeneity of SCW in the gas-like region. The presence of inhomogeneous patterns in the density is evident mainly in the gas-like phase (see Fig. 2a,b) which is in consensus with the absence of the 2^nd^ peak in the radial distribution function (see Fig. 2c) suggesting the loss of the tridimensional tetrahedral structure^42^. The latter was also confirmed by the hydrogen bond distribution analysis. With the increase of temperature the height of the 1^st^ peak increases due to the low density cluster formation which start during the destruction of the hydrogen bond networks^32^.

### Hydrogen bonds

**Figure 3:**
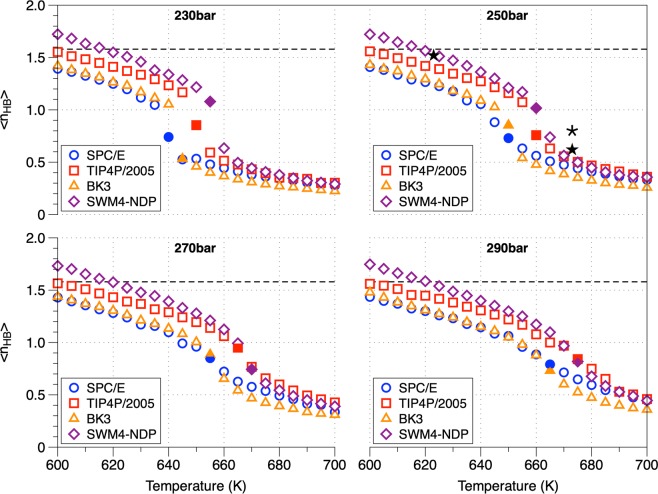
Average number of hydrogen bonds (HB) per water molecule along the studied isobars as a function of temperature. The dashed lines equal to 1.58 indicates the percolation threshold network limit. The *^46^ and ⋆^47^ symbols correspond to experimental data at a studied pressure of 250 bar. The filled symbols are part of the Widom line marking the transition from liquid-like to gas-like behavior.

**Figure 4:**
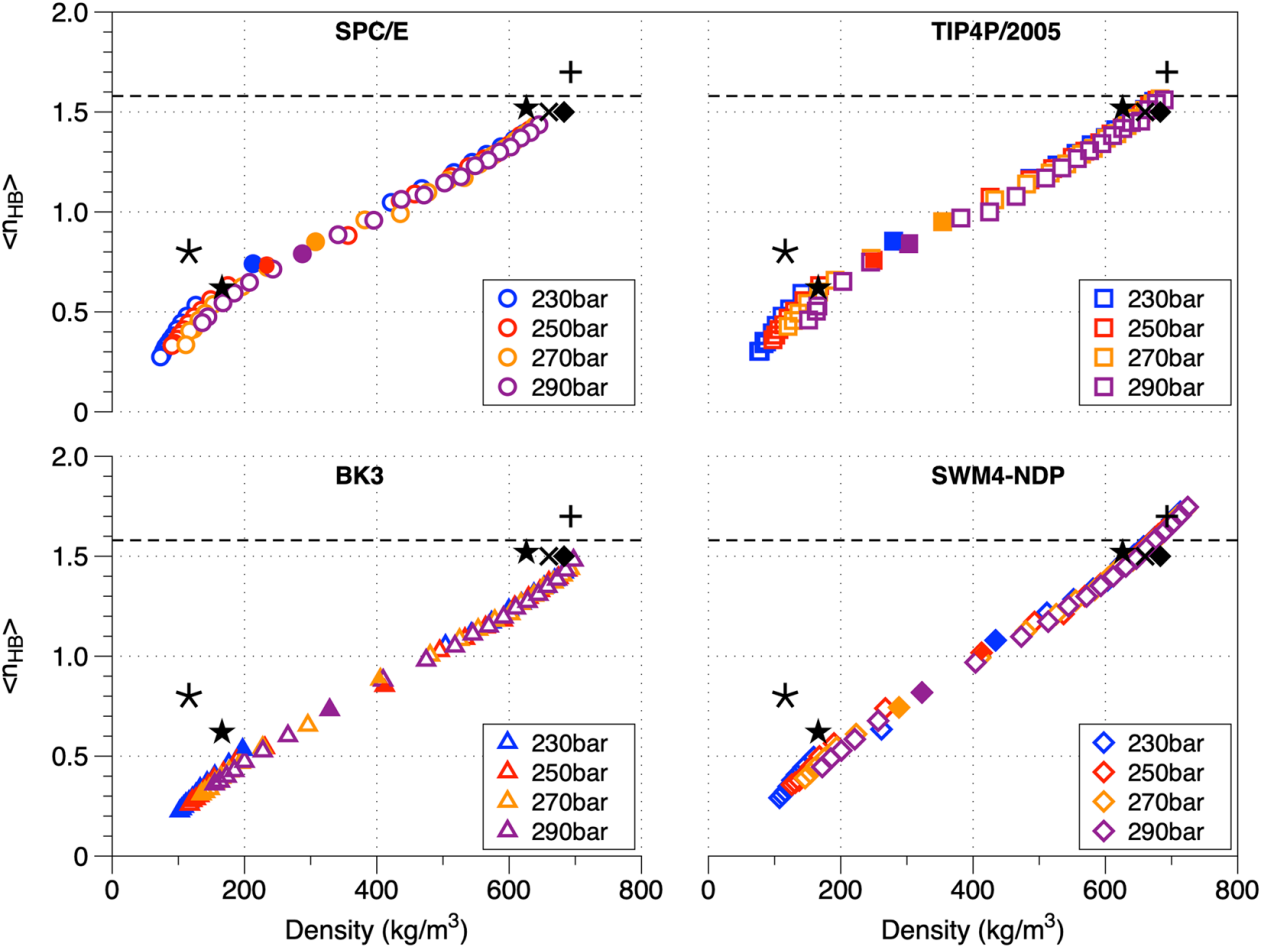
Average number of hydrogen bonds (〈*n*_*HB*_〉) per water molecule as a function of density. The dashed lines equal to 1.58 indicates the percolation threshold network limit. The filled symbols denote the Widom points. The symbols correspond to experimental result which correspond to the specific density but in different pressure range (from 250 to 1000 bar). The symbols *^46^, ⋆^47^ and ×^48^ refer to a pressure of 250 bar while ◆^30^ and +^34^ refer to 1000 bar.

In the current study, a geometric criterion based on the oxygen-oxygen distance (d_OO_ < 3.5 Å) and hydrogen-oxygen-hydrogen angle ($\widehat{HOH}$<30°) has been used to define the hydrogen bonds^42–44^. In the supercritical regime, hydrogen bonds persist (at least up to 800 K) although the hydrogen bonds network is substantially altered (percolation threshold below 1.58)^30–35^. The percolation threshold is a thermodynamic state beyond which no connected hydrogen-bonded chains but only small clusters (down to dimers) of water molecules exist^43,45^. Figure 3 portrays the average number of hydrogen bonds per water molecule (〈*n*_*HB*_〉) as a function of the temperature for the studied pressures. With increasing temperature, a progressive decrease of the 〈*n*_*HB*_〉 occurs, asymptotically approaching zero at higher temperatures^30,35^. The 〈*n*_*HB*_〉 in temperatures above T_c_ (647.17 K) are always below the percolation threshold indicating the absence of a continuous network of hydrogen bonds^30,35,46^. In the case of TIP4P/2005 at T = 600 K and P = 230 bar, the fraction of water molecules with *i* hydrogen bonds (*i* = 0, 1, 2, 3, 4) is f_0_ = 18.4, f_1_ = 34.4, f_2_ = 28.9, f_3_ = 13.9, f_4_ = 4.4 while at T = 700 K is f_0_ = 71.4, f_1_ = 22.3, f_2_ = 5.4, f_3_ = 0.8, f_4_ = 0.1 suggesting that even at high temperatures some degree of hydrogen bonding is still present in the form of dimers and trimers^30^. At higher pressures the fraction of dimers and trimers reduces. Based on the distribution function of monomers and oligomers between these two thermodynamic states (600 K and 700 K respectively), a clear distinction in supercritical water between liquid-like and gas-like phase is evident. The increase of the average number of unbounded molecules (monomers) and the decrease of the “gel” molecules (〈*n*_*HB*_〉 > 1) suggest that SCW forms a inhomogeneous fluid with a sheet-like structure in the gas-like region^2,30,31,35,42,46^. In Fig. 4, the average number of hydrogen bonds per water molecule as a function of density for the studied pressures is portrayed. For all water models, a linear decrease of the average hydrogen bonds in respect to density was identified in both liquid-like and gas-like regions, which allows us to compare the results with data obtained at different thermodynamic conditions (measurements made at elevated pressures)^30,34,46–48^. Overall, the average hydrogen bonds calculated using TIP4P/2005 have a perfect agreement with the experimental data. In the high densities region all FFs are in very good to perfect agreement with the experimental data which is worse in the low-density region (due to the over estimation of the respective densities, see Fig. 1). The number of hydrogen bonds divided by the density (〈*n*_*HB*_〉/*ρ*) in respect to density was calculated suggesting a decrease in respect to the density (Supplementary Information, Fig. S2). A more inhomogeneous behaviour in the gas-like state is observed^45^.

### Static dielectric constant (ε)

**Figure 5:**
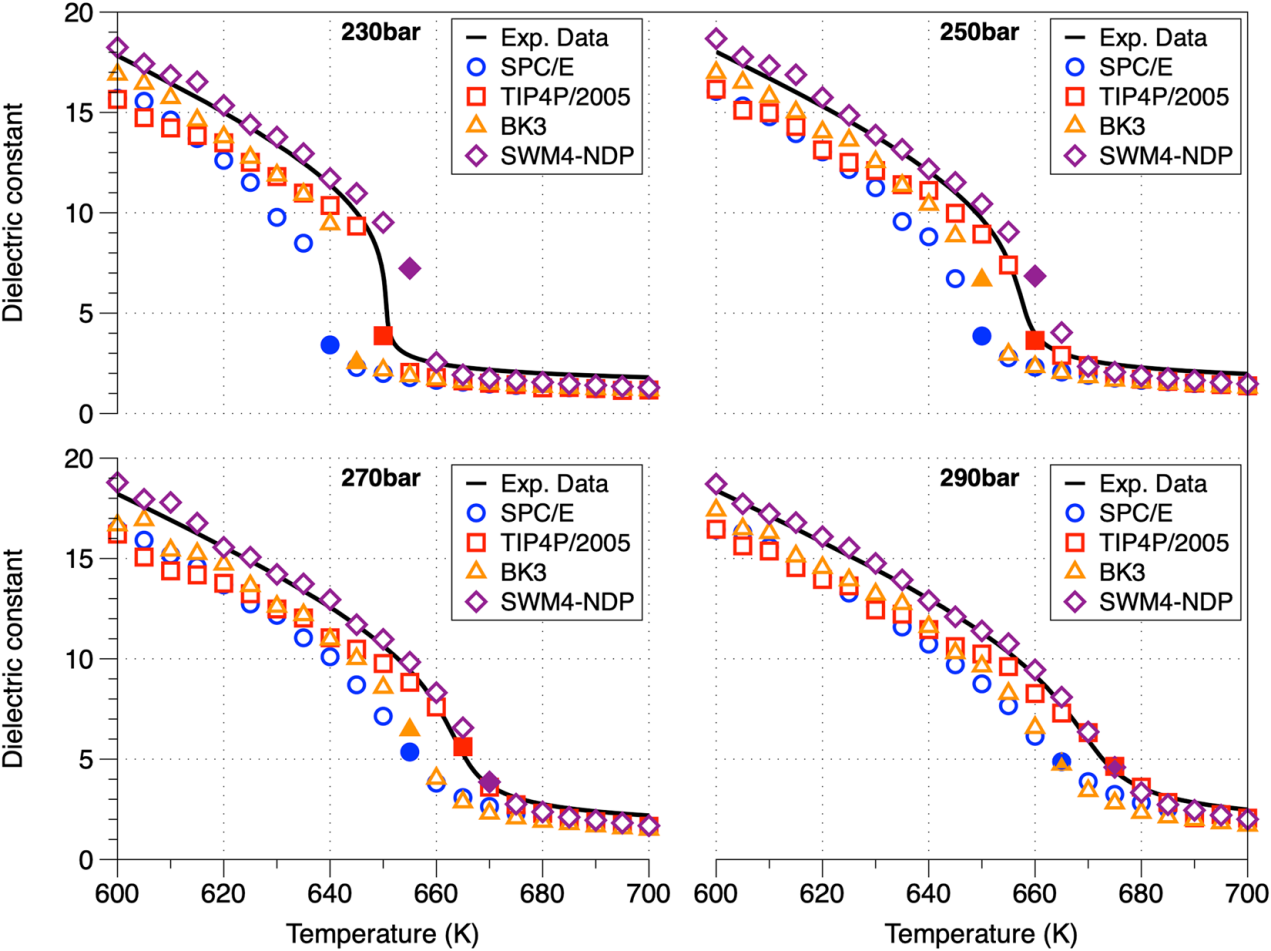
Static dielectric constant along the studied isobars as a function of temperature (standard deviation ± 0.2). The experimental data refer to data obtained by Fernandez *et al*. (1997) which are valid for temperatures and pressures up to 873 K and 10000 bar respectively^37^. The filled symbols describe the Widom points (temperature at which we have maximum of the heat capacity.

One of the most important physical properties for the use of SCW in industrial applications (e.g. in biomass processing) is the dielectric constant which refers to the ability of SCW to dissolve polar or non-polar compounds. In comparison with the density, the dielectric constant refers to the long-range correlations between molecules^49^. The dielectric constant at different pressures as a function of temperature is shown in Fig. 5 and compared with the only available experimental data of Fernandez *et al*.^37^. The simulation results using the polarizable interatomic potentials are in excellent agreement with experimental data under all thermodynamic conditions due to its use of multiple polarization sites^37^. In contrast, the dielectric constant is noticeably under predicted by both the SPC/E and TIP4P/2005 potentials (also at ambient conditions low static dielectric constants and dipole moments are predicted^50^), with the SPC/E potential yielding a typical discrepancy of approximately 10% in the lower temperature regime. Even the dielectric constant is one of the properties that rigid non-polarizable models cannot predict accurately (it is not possible to reproduce both the cohesion energy and the polarization of a certain configuration)^51^, all water interatomic potentials predicted the transition from a liquid-like to a gas-like solvent behavior. The small values in the dielectric constant (<5) in the gas-like region suggest that SCW is an excellent solvent for non-polar molecules. The increase of the solubility of non-polar solutes in SCW (increased ability of SCW to solubilize non-polar compounds) while increasing the pressure was correctly captured (i.e. using the SWM4-NDP water model at 700 K, the dielectric constant at 230 and 290 bar is 1.28 and 2.00, respectively).

### Self-diffusion coefficient (D)

**Figure 6:**
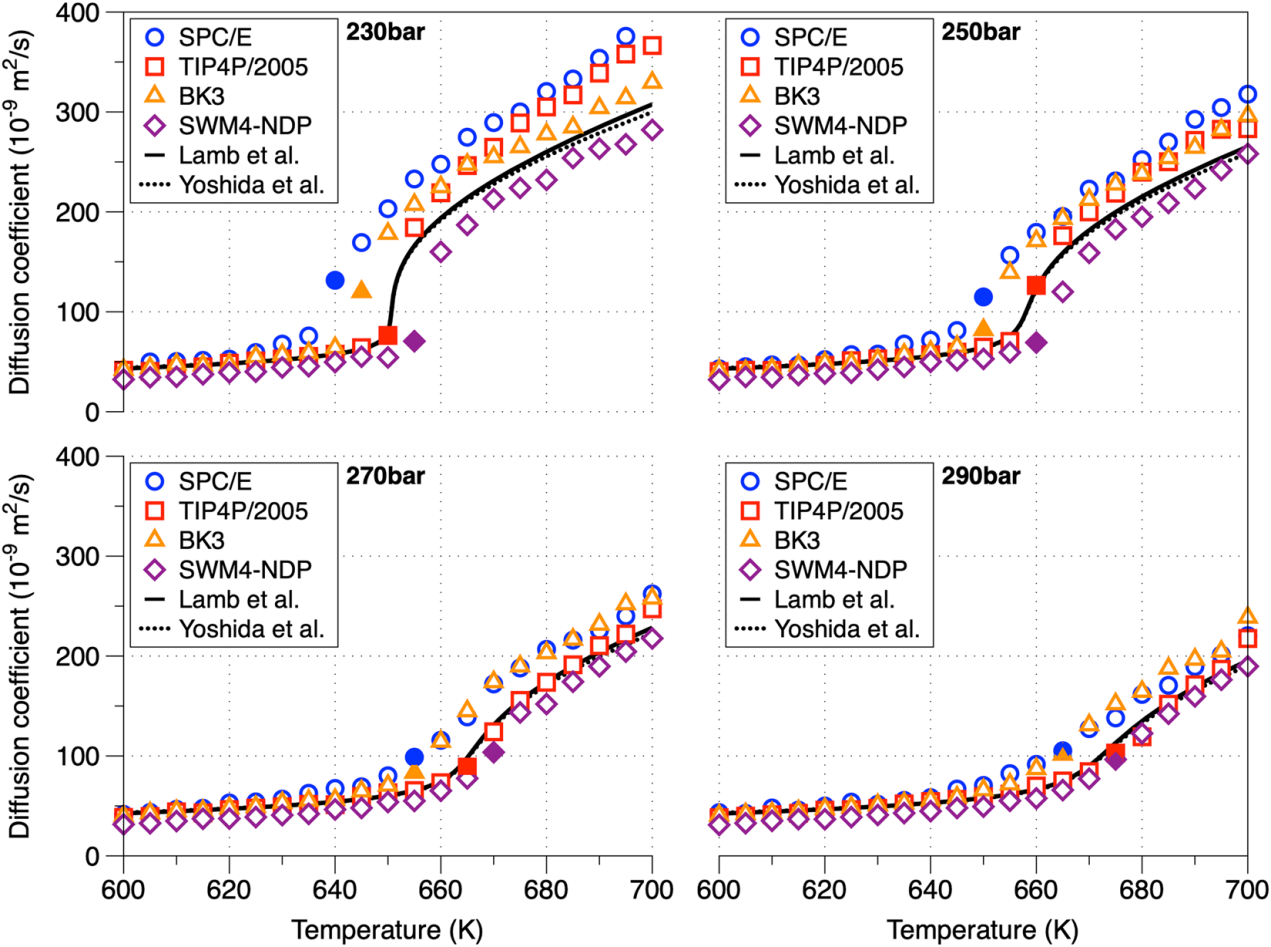
Self-diffusion coefficient of water at different isobars in respect to temperature. Based on experimental data fitting, Lamb *et al*.^52^ managed to correlate the self-diffusion coefficient with the temperature and the density using the equation *D* = 2.24 · 10^−6^ · *T* ^0.763^/*ρ* where *D* is the self-diffusion coefficient (cm^2^/s), *T* is the temperature (K) and *ρ* is the density (g/cm^3^). Yoshida *et al*.^53^ use a function based on the scaling to the hard-sphere model. The Widom points are described by filled symbols.

The self-diffusion coefficients of SCW using multiple water interatomic potentials are compared with the experimental data reported by Lamb *et al*.^52^ and Yoshida *et al*.^53^ in Fig. 6. By comparing the self-diffusion coefficients in respect to temperature (Fig. 6), SPC/E water model consistently overestimates the experimental data with the highest discrepancies in the gas-like regime. The drawback of BK3 model on predicting slightly higher self-diffusion coefficients at ambient conditions^50^ was also captured in the supercritical state. In the isobars of 230 and 250 bar, TIP4P/2005 overestimates the diffusion coefficients, while the remaining isobars are in perfect agreement. By analyzing the self-diffusion coefficients with respect to density, the four isobars collapse in a single curve (in almost excellent agreement with experimental data, see Fig. S3), indicating that density is the main factor governing diffusion^35^. Overall, all water models predict accurately the self-diffusion coefficient at lower temperatures (liquid-like water behavior) while this agreement is worsened at higher temperature (smaller densities) with the temperature and density trend dependence captured. The self-diffusion activation energy (e.g. energy required for breaking the hydrogen bonds of pure water at ambient conditions, 18.8 kJ/mol^54^) in both liquid-like and gas-like regions was determined with the use of the Arrhenius formula $D={D}_{0}{e}^{-{E}_{A}/{k}_{B}T}$ where *D* is the self-diffusion coefficient *E*_*A*_ is the activation energy, *k*_*B*_ is the Boltzmann constant and *T* is the temperature^18^. Table 1 presents the activation energies (kJ/mol) as calculated from the fitting of the aforementioned equation. In the formula, the coefficient *D*_0_ was equal to the calculated self-diffusion coefficient at ambient conditions (varying between the different FFs). In the gas-like region the pressure increase lead to an increase of activation energies which is in consistence with the increase of the number of hydrogen bonds while in the liquid-like region the pressure increase doesn’t significantly affect the activation energies. Along isobars all water models predict activation energies in the same range; the smaller activation energies in the gas-like regime in comparison with the liquid-like regime is attributed to the density decrease.

**Table 1:** The self-diffusion activation energies (kJ/mol) in the liquid like and gas like regions along all isobars studied with maximum statistical uncertainty of 0.2%.

Model	Liquid like				Gas like			
	230 bar	250 bar	270 bar	290 bar	230 bar	250 bar	270 bar	290 bar
SPC/E	31.5	31.7	31.7	32.0	24.6	25.8	27.1	28.1
TIP4P/2005	31.2	31.3	31.5	31.6	23.7	25.1	26.5	27.5
BK3	31.8	32.0	32.0	32.0	25.2	26.0	26.8	27.6
SWM4-NDP	33.0	33.1	33.2	33.2	26.0	26.9	27.7	28.4

## Conclusions

Classical MD simulations using multiple FFs (polarized and polarizable) have been performed in order to study the anomalous behaviour of SCW for a wide range of thermodynamic conditions. The transition from liquid-like to gas-like was captured by analysing the structural characteristics and the physical properties. The absence of the 2^nd^ peak in the oxygen-oxygen RDF indicated the SCW inhomogeneity which was intensified in the gas-like phase. The temperature increase lead to the increase of the 1^st^ peak in the oxygen-oxygen RDF suggesting the cluster formation due to the destruction of hydrogen bond network. In higher temperature regimes, an increase of the monomers fraction indicated a sheet-like structure which yield to an increased ability of dissolving non-polar compounds. The collapse into a single curve of the number of hydrogen bonds and self-diffusion coefficients in respect to density indicated that the main factor governing diffusion is the density. The latter gave the ability to compare the aforementioned results with available experimental data from other thermodynamic conditions. Only the polarizable models (BK3 and SWM4-NDP) estimated with high-accuracy the dielectric constant due to use of multiple polarization sites.

## Methods

### Molecular dynamics simulations

Molecular Dynamics simulations of supercritical water at different thermodynamic conditions (state points) were performed using GROMACS v.2016–4 code^55^. The supercell was consisting of 2048 water molecules. Four different isobars (230, 250, 270 and 290 bar) were examined with a temperature range from T = 600 K to T = 700 K with ΔT = 5 K. The equilibrium runs were performed in the isothermal-isobaric (NPT) ensemble and the sampling runs were performed in both NPT and canonical (NVT) ensembles. The length of each sampling run was 10 ns. The equations of motions have been integrated using the leap-from algorithm with an integration time step of 1 fs, to ensure energy conservation. The temperature was controlled using a Nosé-Hoover thermostat with a relaxation time of 1 ps and the pressure was controlled using an isotropic Parrinello-Rahman barostat with a relaxation time of 1 ps. The particle-mesh Ewald method has been used to evaluate the long-range electrostatic interactions with a cut-off of 1.4 nm. The water interatomic potentials are not able to reproduce the critical temperature and pressure (associated with the under/over estimation of the vaporisation enthalpy); consequently an offset in the temperature and pressure was applied (i.e. in the case of TIP4P/2005 water model the simulated temperature of 640 K corresponds to the experimental 647.1 K – offset of approximately 7.1 K). The critical parameters of the water models are presented in Table 2.

**Table 2:** Critical parameters (temperature, pressure and density) of the water models and of experimental water.

System	T_c_ (K)	P_c_ (bar)	ρ_c_ (g/cm^3^)
SPC/E	638.6	139	0.27
TIP4P/2005	640	146	0.31
BK3	634	214	0.32
SWM4-NDP	576	199	0.32
Experiment	647.1	220.6	0.32

### Water interatomic potentials

MD simulations of supercritical water have been performed using non-polarizable SPC/E^56^, TIP4P/2005^57^ and polarizable SWM4-DP^58^ and BK3^40^ interatomic potentials. The intermolecular pair potential used for all force-fields, has two contributions, a 12-6 Lennard-Jones term and an electrostatic interaction term. In the BK3 force-field, a charge-on-spring model using Gaussian charges has been implemented while in the remaining force-fields constant charges were used. The non-electrostatic interactions were described using a Buckingham term.

### Determination of physical properties

#### Bulk-density (ρ)

The density *ρ* at constant pressure (isothermal-isobaric ensemble, NPT) follows from the mass, *m* of the system divided by its mean volume V

$$\rho =\frac{m}{\langle V\rangle }$$

where the bracket denotes time averaging over the simulation period^59^.

#### Static dielectric constant (ε)

The expression of the static dielectric constant (relative static permittivity), ε, is given by

$$\varepsilon ={\varepsilon }_{\infty }+\frac{\langle {{\rm M}}^{2}\rangle -{\langle {\rm M}\rangle }^{2}}{3{\varepsilon }_{0}V{k}_{B}T}$$

where the angle brackets denote time averaging over the simulation period, *ε*_*∞*_ is the dielectric constant at optical frequencies (in rigid non-polarizable molecules it is equal to unity, 1^29^), *V* is the volume, T is the temperature and M is the total dipole moment $({\rm M}={\sum }_{i}^{N}{\mu }_{i})$^60^.

In the case of polarizable molecules

$$\frac{{\varepsilon }_{\infty }-1}{{\varepsilon }_{\infty }+2}=\frac{4\pi \alpha }{3\langle V\rangle }$$

where *α* is the polarizability $(\alpha ={q}_{D}^{2}/{k}_{D})$) and $\langle V\rangle$ is the mean volume. The spring constant (*k*_*D*_) was assumed equal to 1000 kcal/mol/Å^2^ which ensures that the point-dipole limit is valid^61^.

#### Self-Diffusion Coefficient (D)

The self-diffusion coefficients were obtained from simulations in the NVT ensemble. For each atomic constituent, the mean square displacement (MSD) - which indicates the average displacement of a tagged atom during a fixed time t, calculated by summing the square of distance over all the atoms and dividing by the number of atoms, N, as follows^59^

$$MSD=\langle {\Delta }^{2}r(t)\rangle =\frac{1}{N}\mathop{\sum }\limits_{i=1}^{N}\langle {[{r}_{i}(t)-{r}_{i}(0)]}^{2}\rangle $$

where *r*_*i*_(*t*) is the position of atom i at time t. The angular brackets indicate an average over the positions of the atoms at time t = 0. The coefficient of self-diffusion of a particle may be obtained from the MSD for sufficiently long simulation times (over 10 ns) by use of the Einstein equation

$$D=[\frac{1}{6t}]\langle {\Delta }^{2}r(t)\rangle $$

#### Isobaric heat capacity (c_p_)

By using the enthalpy fluctuation formula, the isobaric heat capacity was computed according

$${c}_{p}={(\frac{\vartheta H}{\vartheta T})}_{p}=\frac{\langle {H}^{2}\rangle -{\langle H\rangle }^{2}}{{k}_{B}{\langle T\rangle }^{2}}$$

#### Pair correlation functions

The short-range order of water molecules was described via the Radial Distribution Function (RDF), symbolized as *g*(*r*) and expressed as^59^

$${g}_{ij}(r)=\frac{1}{\rho }\frac{n(r)}{V}=\frac{V}{{N}_{i}{N}_{j}}\sum _{j}\frac{n(r)}{4\pi {r}^{2}\Delta r}$$

where *g*_*ij*_ denotes the partial RDF of the i^th^ and j^th^ atom species, *N*_*i*_ and *Nj* are the numbers of the species i and j, V is the volume of the system, and *n*(*r*) denotes the average number of the ions j surrounding ion i in a spherical shell defined by radii *n*(*r*) ± Δ*r*/2.

## Supplementary information

Supplementary information
