# Supplementary material for: Supercritical water anomalies in the vicinity of the Widom line
Source: Sci Rep. 2019 Oct 31;9:15731. doi: 10.1038/s41598-019-51843-0 (PMC6823507; doi:10.1038/s41598-019-51843-0)
Supplement: Supplementary file 1 — Supplementary information [file 41598_2019_51843_MOESM1_ESM.pdf]

Supercritical water anomalies in the vicinity of the Widom line

Konstantinos Karalis[1*], Christian Ludwig[2,3], Bojan Niceno[1,4*]

[1]Laboratory for Scientific Computing and Modelling (LSM), NES Division, Paul Scherrer Institute, 5232, Villigen PSI, Switzerland

[2]Laboratory for Bioenergy and Catalysis (LBK), ENE Division, Paul Scherrer Institute, 5232, Villigen PSI, Switzerland

[3]École Polytechnique Fédérale de Lausanne (EPFL), ENAC IIE GR-LUD, 1015, Lausanne, Switzerland

[4]Eidgenössische Technische Hochschule Zürich (ETHZ), MAVT-LKE, 8092, Zurich, Switzerland

Supplementary Information

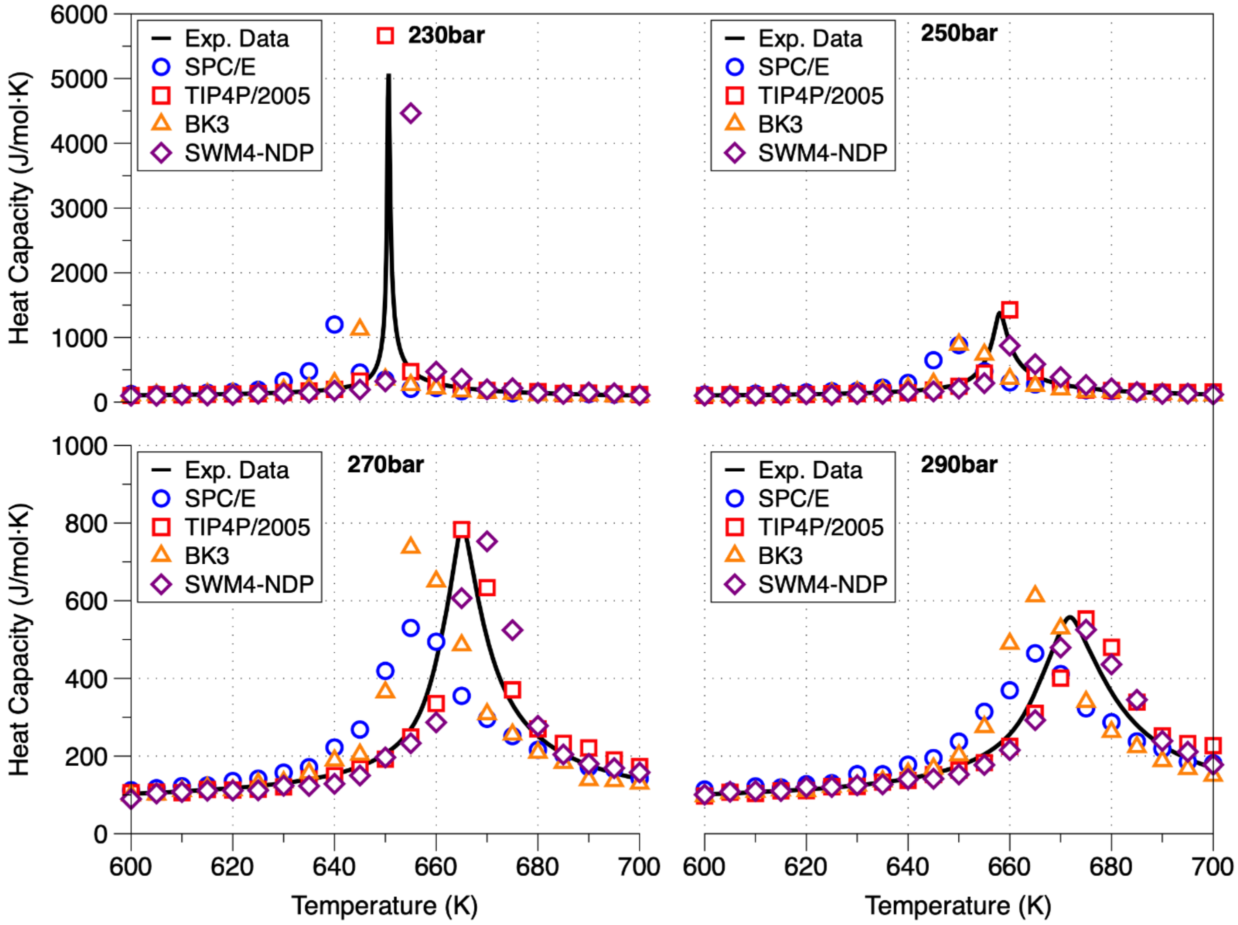

Figure S1. Heat capacity of water at different isobars in respect to temperature. Based on the maxima of the heat capacity, the Widom line was determined.

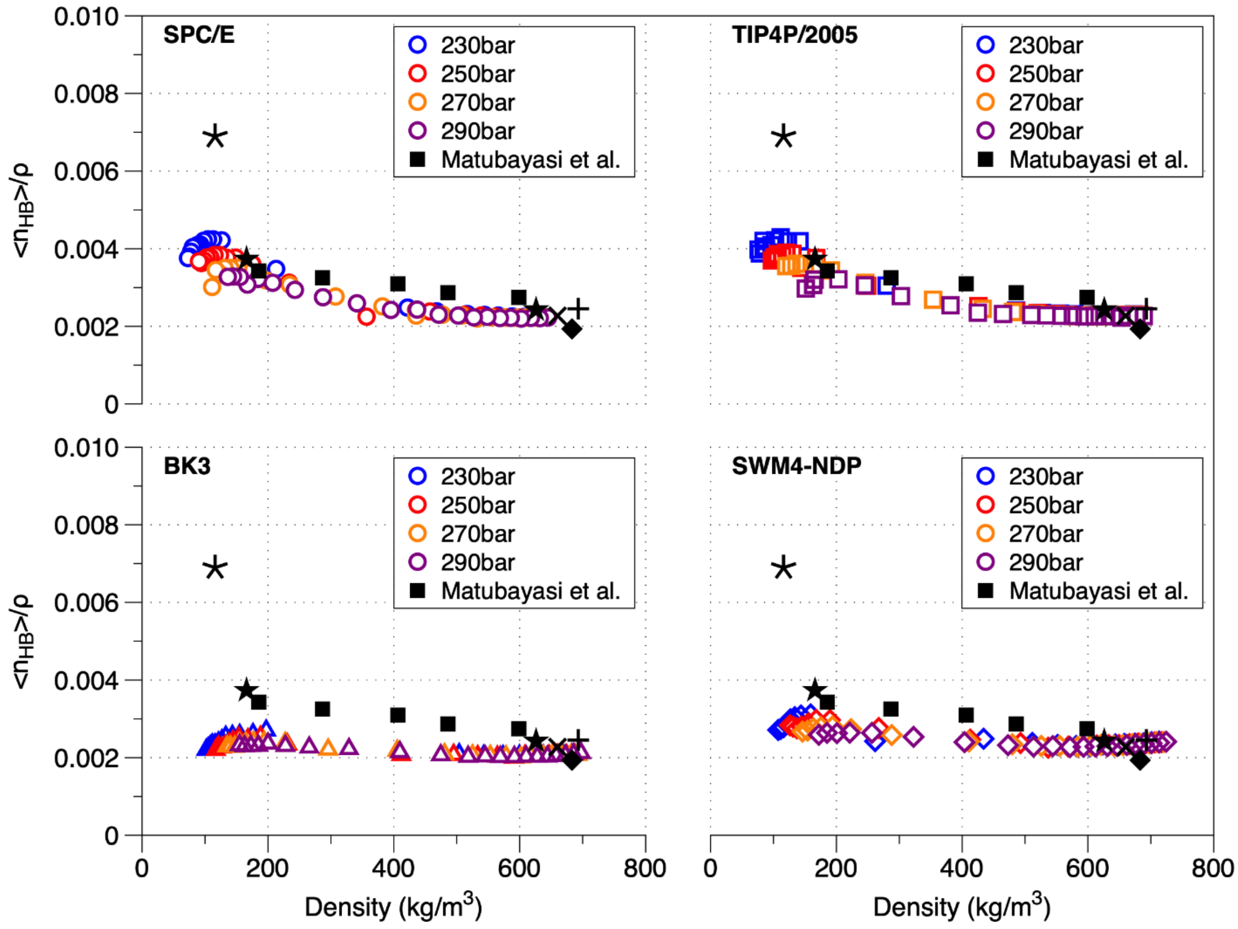

Figure S2. The number of hydrogen bonds divided by density ($< n_{HB} >/rho$) in respect to density (rho). The filled symbols denote the Widom points. The symbols correspond to experimental result which correspond to the specific density but in different pressure range (from 250 to 1000 bar). The symbols ⋆ [47], ★[48] and ✕ [49] refer to a pressure of 250bar while ◆[29] and +[33] refer to 1000 bar.

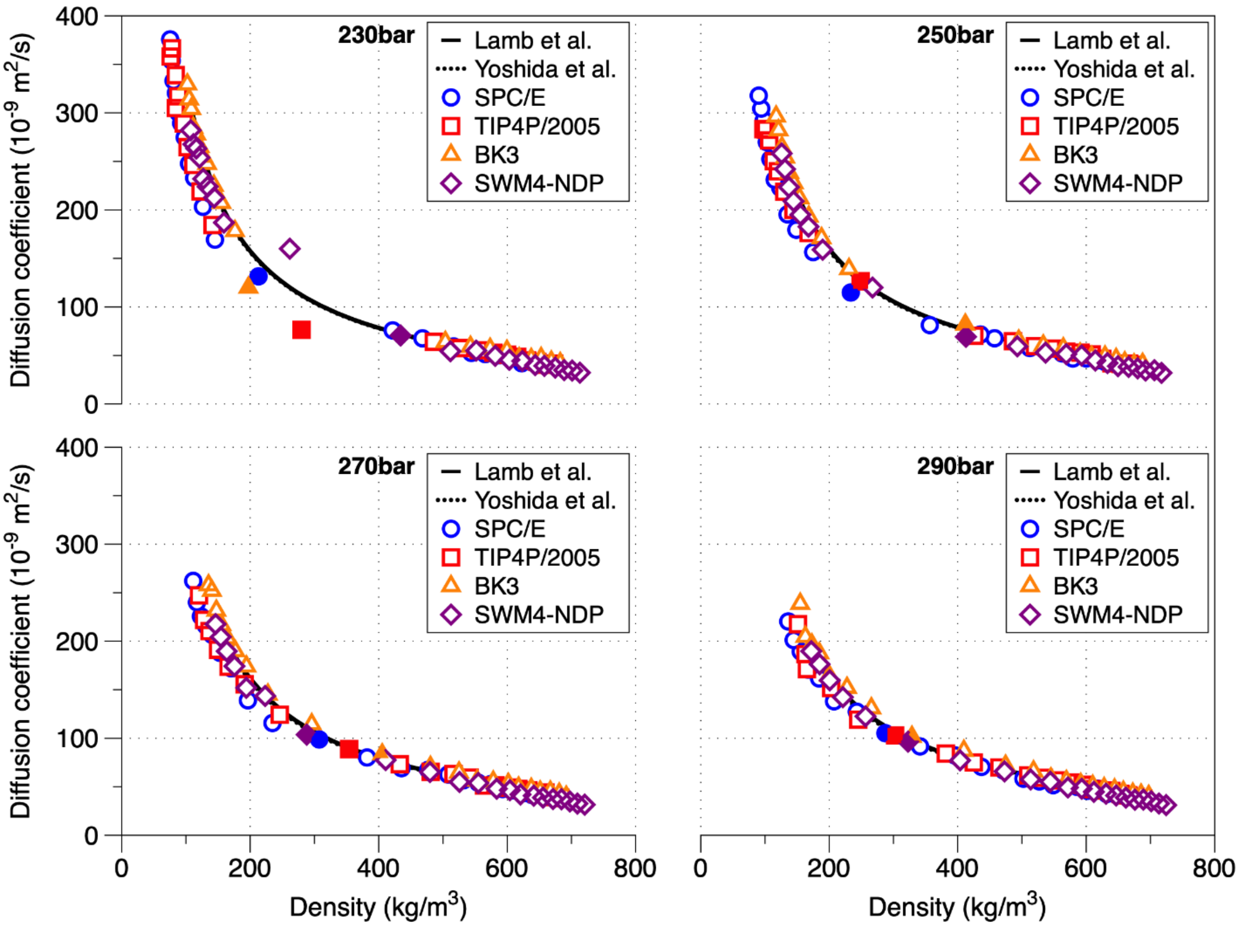

Figure S3. Self-diffusion coefficients in respect to density. The filled symbols denote the Widom points.
